# Enhancing mentalization by specific interventions within mentalization-based treatment of adolescents with conduct disorder

**DOI:** 10.3389/fpsyg.2023.1223040

**Published:** 2024-01-08

**Authors:** Lea A. Kasper, Sophie Hauschild, Lisa M. Schrauf, Svenja Taubner

**Affiliations:** ^1^Institute for Psychosocial Prevention, University Hospital Heidelberg, Heidelberg, Germany; ^2^Psychological Institute, University of Heidelberg, Heidelberg, Germany

**Keywords:** mentalization, reflective functioning, in-session, interventions, conduct disorder, psychotherapy process

## Abstract

**Objective:**

Mentalization is discussed as a mechanism of change in psychotherapy due to its positive effects on psychological functioning. In order to specifically apply mentalization-based interventions, a better understanding of the relationship between interventions and in-session mentalization is needed. The study aimed to explore the association between interventions and effective mentalizing.

**Method:**

Fifteen therapy sessions of three therapies with male adolescents with conduct disorder were transcribed and rated with the Reflective Functioning (RF) Scale and a newly developed Mentalization-based Treatment (MBT) intervention coding manual. The coded interventions were categorized into intervention levels according to the MBT manual. Fisher’s exact tests were performed to test differences in frequencies of interventions in high-RF sequences (RF score ≥ 4) compared with remaining therapy sequences (RF score ≤ 3).

**Results:**

Specific MBT interventions such as demand questions, affectelaboration, empathic validation, change of subject, challenge, patienttherapist relation and mentalizing for the patient were related to effective mentalizing. Moreover, intervention levels such as supportive & empathic, basic- mentalizing & affect mode and relational mentalizing were positively associated with effective mentalizing.

**Conclusion:**

MBT interventions seem to promote effective mentalizing at various intervention levels. Interventions that enhance effective mentalizing seem to be patient specific. In line with MBT theory, their effect on effective mentalizing might depend on various variables, such as the patients’ arousal and pre-mentalizing mode.

## Introduction

### Conduct disorder

Conduct Disorder (CD) is described as repetitive and chronic patterns of aggressive behavior toward people, animals, or other people’s property, norm-violating behavior, and cheating or stealing according to the Diagnostic and Statistical Manual of Mental Disorders-5 (DSM-5; American Psychiatric Association, 2013). The global prevalence of CD in youth is estimated to be 2 to 10% with an increased prevalence in boys compared to girls (Costello et al., 2003; Ravens-Sieberer et al., 2008; Petermann and Petermann, 2013; Polanczyk et al., 2015). In addition, the likelihood of people with CD developing antisocial personality disorder (ASPD) is increased (Ridenour et al., 2002; Lahey et al., 2005; Pardini and Frick, 2013). ASPD is characterized by antisocial behavior, delinquency and recklessness as well as a lack of empathy, a lack of guilt and an inability to maintain relationships (Vloet et al., 2006).

Empirical studies have found evidence for reduced mentalizing abilities in adolescents with disorders of conduct and emotions (Cropp et al., 2019b) as well as in young and adult violent offenders (Taubner, 2008b; Möller et al., 2014; Newbury-Helps et al., 2017). Mentalizing describes the ability to imagine mental states in one’s self and in other people to explain behavior (Fonagy et al., 2002). In detail, this refers to imagining mental processes, such as thoughts, feelings, desires, beliefs, or needs, which enables individuals to explain and predict behavior to some extent (Fonagy et al., 2002; Allen et al., 2008). Mentalizing was identified as a protective factor against externalizing behaviors such as aggression and delinquency (Taubner et al., 2016; Morosan et al., 2020). Therefore, it is hypothesized that the promotion of mentalizing addresses a fundamental psychopathological mechanism of CD. As a result, Mentalization-based Treatment (MBT) was proposed to be a suitable treatment for individuals with CD (Taubner et al., 2021). Within MBT for adolescents with CD (MBT-CD), a particular focus is put on the development of an understanding of interpersonal situations and emotions, as well as understanding specific triggers and mentalization breakdowns associated with antisocial and aggressive behavior (Taubner et al., 2021). MBT-CD aims to achieve a promotion of adolescents’ emotion regulation and increase their scope of action through enhancing effective mentalizing (Hauschild et al., 2023). However, how effective mentalizing can be promoted during therapy sessions still remains an open question.

### Mentalization in psychotherapy

Overall, MBT specifically aims to maintain an optimal level of emotional arousal to explore feelings and mental states as well as their influence on relationships (Taubner and Sevecke, 2015; Taubner et al., 2019). It is assumed that some activation of attachment, which is closely related to arousal, is necessary for effective mentalizing. If the activation of attachment or arousal is too low or too high, effective mentalizing fails and pre-mentalization sets in (Bateman and Fonagy, 2016). Process recommendations in MBT suggest to interrupt patients’ pre-mentalizing modes, manage patients’ arousal and establish accurate mentalization. Regarding the patients’ arousal level four intervention levels (supportive & empathic; clarification, exploration & challenge; basic-mentalizing & affect mode; relational mentalizing) have been suggested (Bateman and Fonagy, 2016). MBT can be applied to a variety of clinical disorders, but these core principles of MBT remain similar (Lemma et al., 2010; Luyten et al., 2012; Weijers et al., 2016).

Enhanced mentalization is assumed to be related to a general improvement of psychological functioning as lower depression severity, less interpersonal problems, general distress (Levy et al., 2006; Taubner et al., 2011; Ekeblad et al., 2016; Babl et al., 2022). Therefore, mentalization is highly relevant for psychotherapeutic processes and discussed as a mechanism of change (Katznelson, 2014). Effective mentalizing can be defined as the establishment of a new, meaningful connection between cognition and affect that alters intrapsychic functioning and thus enables new behavior (Taubner, 2008a). It can be hypothesized that MBT, with its specific focus on fostering mentalizing skills, establishes effective mentalizing in specific interpersonal contexts and thus contributes significantly to the therapeutic success (Taubner, 2008a). However, the exact mechanisms of change during the process of mentalization remain to be investigated (Volkert et al., 2019).

By focusing on state-like processes, it is possible to examine mechanisms of psychotherapy in more detail (Zilcha-Mano, 2021). Mentalizing measured within therapy sessions seems to correspond to a personality state, whereas mentalizing measured with the Adult Attachment Interview (George et al., 1996) depicts an enduring and more difficult to change personality trait (Hörz-Sagstetter et al., 2015). Consistent with this, it was found that mentalizing fluctuates strongly within therapy sessions, particularly in association with therapeutic interventions (Hörz-Sagstetter et al., 2015; Möller et al., 2017; Kornhas et al., 2020; Kivity et al., 2021; Meier et al., 2023). It is emphasized that future studies should examine which interventions specifically enhance mentalizing (Hörz-Sagstetter et al., 2015).

### Mentalization enhancing interventions

To the best of our knowledge, only three sub-analyses of randomized controlled trials and two controlled case studies examined the association between mentalization enhancing interventions and mentalizing within therapy sessions so far (Möller et al., 2017; Georg et al., 2019; Kornhas et al., 2020; Kivity et al., 2021; Meier et al., 2023). In these studies, mentalization was measured with the Reflective Functioning Scale (RF Scale, Fonagy et al., 1998) for each statement within the patients’ speech (Möller et al., 2017; Georg et al., 2019; Kivity et al., 2021) or for each three-minutes segment of the therapy session based on the patient’s statements within these segments (Kornhas et al., 2020; Meier et al., 2023).

Möller et al. (2017) investigated whether the therapists’ use of interventions that are fundamental to MBT was associated with patients’ mentalizing in psychotherapy sessions. Therapy transcripts of 15 patients with two psychotherapy sessions each were used. Frequency and quality of interventions critical to MBT as assessed with the MBT adherence and competence scale (Karterud et al., 2013) were positively associated with mentalizing within therapy sessions. In addition, the authors classified therapists’ statements as demanding mentalizing (*“why do you think your boyfriend said that?”*) or permitting mentalizing (*“tell me more about what you did around that time.”*) and found that the use of demand questions increased mentalizing in patients’ immediate responses (Möller et al., 2017).

Building on this, Kivity et al. (2021) analyzed 205 transcripts of psychotherapy sessions from 88 patients. Demand questions resulted in increased mentalizing in the patients’ immediate responses compared to the permit questions. Moreover, demand questions had a down-regulatory effect on patients’ arousal (Kivity et al., 2021).

Limiting the analyses not only to demand questions, Meier et al. (2023) conceptualized 40 mentalization enhancing interventions referring to four MBT principles (process, not knowing stance, affect focus and relationship). To assess the relation between the mentalization enhancing interventions and mentalization, 84 therapy sessions from 28 patients were analyzed. The frequency of mentalizing enhancing interventions in proportion to mentalizing non-enhancing interventions was related to the patients’ mentalizing within therapy sessions (Meier et al., 2023). However, only using the proportion of mentalizing enhancing interventions no conclusion about the influence of individual interventions could be drawn. It can only be concluded that the theoretically conceived interventions strengthen mentalizing, but not which of these 40 mentalization enhancing intervention is particularly helpful.

To investigate the relation between demand questions, content themes and mentalization over the course of a long-term therapy with a patient with Borderline personality disorder, 20 therapy sessions at five time points were randomly selected (Kornhas et al., 2020). As expected, demand statements resulted in the patients’ higher mentalizing responses. Furthermore, the patient’s statements per three-minute segment were divided into important recurring themes. Despite its essential importance within MBT, the patient-therapist relationship was rarely discussed.

In a case study of focused parent-infant psychotherapy with a depressed mother, relevant moments during patient’s increased mentalizing were analyzed (Georg et al., 2019): Three interventions such as “supporting the parent by means of offering psychological functions (e.g., mentalizing for him/her or structuring),” “encouraging the parent to report on significant themes, events or experiences” and “perceiving and verbalizing the affective quality in the observed relationship” were most frequently observed in effective mentalizing sequences.

From the perspective of adolescent patients with CD first indications regarding the acceptance of individual interventions could be found (Hauschild et al., 2021). With the help of the qualitative analysis of therapy evaluation interviews it became apparent that the patients appreciated “having someone to talk to.” They also found it helpful to gain new perspectives and to reflect on their own behavior. Patients stated that they gained “more self-control through improved insight.” At the same time, some patients in the study found the questioning technique irritating. Therefore, it is particularly relevant to understand which interventions within the treatment of adolescents with CD can be considered helpful in terms of increasing mentalization.

### Aim of the study

The importance of better understanding the process of mentalization within the therapy has been pointed out repeatedly (Hörz-Sagstetter et al., 2015; Volkert et al., 2019). For this, the question of the association between therapeutic interventions and change in mentalization is important. Initial studies have shown that interventions can strengthen mentalization. On the one hand, a large number of interventions were combined and tested (Meier et al., 2023), whereby no conclusions can be drawn about specific interventions. On the other hand, the intervention of the demand question to strengthen mentalization was emphasized several times (Möller et al., 2017; Kornhas et al., 2020; Kivity et al., 2021). Aside from demand questions, no specific other interventions have been examined with regard to their direct relation to mentalization except for a case study on parent infant therapy (Georg et al., 2019). Thus, a variety of interventions central to MBT have been neglected in empirical studies. It cannot be assumed that the intervention demand question can solely improve mentalization. Furthermore, to the authors’ knowledge, there are no data on the design of interventions over the course of therapy, whether, for example, certain phases of intervention or intervention level can be identified.

In the current study, two research questions will be addressed in an explorative approach:

(1) Which intervention levels (supportive & empathic; clarification, exploration & challenge; basic-mentalizing & affect mode; relational mentalizing) are used in MBT-CD over the course of therapy?
(2) Which specific interventions and intervention levels are related to enhance effective mentalizing throughout the therapeutic process in MBT-CD?

To answer these research questions a comprehensive qualitative and quantitative approach is required. In order to implement the research project, we selected a small number of patients and a relatively sizeable number of therapy sessions per patient in order to be able to map the course of therapy. In this way, the change in intervention levels over the course of therapy can be illustrated. To identify the wide range of used interventions in therapy sessions an inductive and deductive approach was taken. The interventions were coded statement by statement using verbatim transcript analogous to the RF coding procedure in order to observe associations between them.

A multi-patient case study was chosen to capture the uniqueness of interventions and to identify patterns between interventions and mentalization across patients. This study is intended to generate initial hypotheses about the use of interventions and intervention levels over the course of therapy as well as the association of interventions and mentalization in an exploratory approach.

## Methods

### Procedure

The psychotherapy cases used for the current study were part of a feasibility and pilot study for MBT-CD (Hauschild et al., 2023). The study was approved by the Ethics Committee of the Heidelberg University Medical Faculty (Germany; S-534/2016) and registered at clinicaltrials.gov (NCT02988453). The study design is presented in detail in Taubner et al. (2021). Three patients treated by the same therapist were chosen from the trial: on the one hand, because they were characteristic for the MBT-CD target group in terms of typical symptoms (lying, violence) and showed a successful outcome in terms of change in diagnosis and, on the other hand, to reduce therapist variance and to build kind of a prototype for MBT-CD by only using data from a highly skilled MBT supervisor and trainer. Per patient, five therapy sessions were selected over the course of therapy: one session at the beginning, three sessions from the middle and one session from the last third of therapy. The selection was based on time points of each therapy session in relation to the total amount of therapy sessions. Therapy sessions were transcribed verbatim using the software F4transcript (autotranscription, version 6.2.6) and randomized between patients and time points to ensure a blinded coding of the patients’ statements with the RF Scale (Fonagy et al., 1998) and of the therapist’s statements with a newly developed intervention coding guide (Kasper et al., 2023) (Supplementary material S1). To analyze moments of effective mentalizing, RF scores (≥ 4) and the associated interventions were used to capture high-RF sequences.

### Therapist

The therapy was provided by an experienced psychotherapist for adults, adolescents, and children with specializations in psychodynamic psychotherapy (ST). The therapist is certified as a supervisor and trainer for MBT by the Anna Freud Centre London (AFC) and developer of MBT-CD. Adherence of therapy sessions to MBT was assessed in four of the total of 15 (26.7%) therapy sessions by using Bateman (2018) Adherence and Competence Scale (MBT-AC). Adherence was existent with an average score of 5.2 according to the MBT-AC manual (Bateman, 2018). Adherence ratings were performed by three raters to confirm inter-rater reliability (SH and two other reliable MBT-AC rater), which was average (Koo and Li, 2016) using a two-way mixed, absolute agreement, with an ICC of 0.72.

### Patients

The patients were three males: Thomas,^1^ 17 years old at therapy start, with a treatment duration of 18 months and 45 therapy sessions; Steven, 16 years old at therapy start, with a treatment duration of 24 months and 59 therapy sessions; Noah, 18 years old at therapy start, with a treatment duration of 22 months and 56 therapy sessions.

Diagnostics were performed pre and post therapy by using the Clinical Interview for DSM-5 (SCID-II; Fydrich et al., 1997) and the Mini-International Neuropsychiatric Interview for Children and Adolescents (Mini-Kid; Sheehan et al., 2010). Thomas fulfilled the criteria for Oppositional Defiant Disorder (ODD) and Attention Deficit Hyperactivity Disorder (ADHD). Steven met criteria for CD and Obsessive-Compulsive Disorder. Noah met the criteria for Borderline and Antisocial Personality Disorder (ASPD).

Further the Global Assessment of Functioning (GAF; Hall, 1995) was collected pre and post treatment. The GAF measures the general level of functioning in the areas of psychological, social, and occupational functioning. It ranges from 1 (lowest level of functioning) to 100 (highest level of functioning). All three adolescents showed a score between 45 and 49 (Thomas: 49; Steven: 46; Noah: 45).

In addition, mentalizing ability was assessed before and after treatment using the Brief Reflective Functioning Interview (BRFI; Rudden et al., 2005). The BRFI is a semi-structured interview designed for the assessment of mentalization with the RF-Scale (Fonagy et al., 1998). The instrument consists of 11 open questions that ask respondents to reflect on their attachment relationships. Thomas showed an RF score of 2 (lacking to low mentalizing), Steven showed an RF score of 3 (low mentalizing) and Noah showed an RF score of 5 (ordinary mentalizing). 2 of the 6 (30.3%) BRFIs were performed by two raters to confirm inter-rater reliability (LK and another reliable RF rater), which was excellent (Koo and Li, 2016) using a two-way mixed, absolute agreement, with an ICC of 1.

### Treatment

MBT-CD included one weekly individual session and one monthly family session over the course of therapy. To provide a personalized treatment, duration and number of individual or family sessions was tailored to each participant. MBT-CD started with two psychoeducational sessions for the adolescent and their family on mentalizing and reciprocal effects of difficulties with mentalizing and handling emotionally challenging situations. MBT-CD is described in more detail in Hauschild et al. (2023).

### Measures

#### The Reflective Functioning Scale (RF scale)

The RF Scale (Fonagy et al., 1998) is an 11-point rating scale for the assessment of mentalizing in the context of attachment relationships. The observer-based scale is applied to transcribed interviews such as the Adult Attachment Interview (George et al., 1996) or the Brief Reflective Functioning Interview (Rudden et al., 2005) or therapy transcripts (RF in-session). “This rating applies to passages in response to demand questions, or whenever (in the rater’s view) there is an implied demand for a mentalizing response to a probe” (Fonagy et al., 1998, p. 28). The quality of mentalizing is coded from-1 to 9 (exceptional RF), whereby −1 means negative RF, 1 means lacking RF, 3 means questionable or low RF, 5 means ordinary RF, 7 means marked RF and 9 means exceptional RF. The Coding is made regarding four dimensions: (1) awareness of the nature of mental states, (2) effort to understand mental states underlying the behavior, (3) recognition of the developmental aspects of mental states, and (4) ability to reflect on mental states in relation to the interviewer or therapist (Fonagy et al., 1998). All ratings of RF were performed by reliable and certified RF raters.

##### RF in session

To capture RF within therapy transcripts, the RF in-session manual (Talia et al., 2015) was designed, which divides the patient’s statements into 150-word sections and scores them for RF. In contrast, in this study each patient statement was rated: per therapy session each statement made by the patients was coded blinded for stage of therapy and patient assignment with the in-session RF Scale (Talia et al., 2015). In psychotherapy, it is the failure to respond to an implicit demand (given by the context of the therapy session) or explicit demand (in a demand question) that can be scored with less than a score of 3. For two of the fifteen (13.3%) sessions, a second RF rating was performed (LK, SH). Inter-rater reliability for the RF score was good (Koo and Li, 2016) using a two-way random, absolute agreement, with an ICC of 0.74–0.85.

##### High-RF sequences

We defined effective mentalizing during therapy as sequences where high RF is present. According to the RF Scale a score below 4 indicates low to negative RF and a score equal and above 4 indicates ordinary to high RF (Fonagy et al., 1998). Thus, all statements with an RF greater than or equal to 4 were analyzed in detail for content and prior interventions. Sequences were chosen as paragraphs that consist of the content that lead to effective mentalizing as documented by the high-RF. All interventions during this sequence were assessed. These sequences are referred to as high-RF sequences and were defined in consensus ratings (LK, LS). Table 1 illustrates a high-RF sequence with its starting and ending point from Thomas. In this example, it is a great achievement for the patient to communicate his own mental states such as convictions and motives to the therapist. However, it is important to emphasize that mental states are attributed to the parents with too much certainty and thus no opaqueness is given.

**Table 1:** Example of a high-RF sequence including the intervention levels and the patients’ RF.

Intervention levels	RF	Therapist/Patient	Statement	High-RF sequence
Supportive & empathic		T:	*“And I would like you to write down what you think and not to write down what you think the others want to hear.”*	
	RF of 1	P:	*“Yes.”*	
Clarification, exploration & challenge		T:	*“Do you think that’s possible? Because based on the sheets you gave me, I had the feeling that there’s a lot on there what the others want to hear and not necessarily what you think.”*	Start
	RF of 3	P:	*“Hm, that’s just how it goes with me, also at home. I say – so when I’m in conflict as well – I say what they want to hear.”*	
Supportive & empathic		T:	*“Yes.”*	
	RF of 1	P:	*“So that everybody agrees like that.”*	
Clarification, exploration & challenge		T:	*“So that there is peace.”*	
	RF of 1	P:	*“Yes.”*	
Supportive & empathic		T:	*“I understand that too.”*	
	RF of 4	P:	*“Because if I then open my mouth against it, then I know that it ends in stress, because then they have not heard what they want to hear.“*	End

#### MBT interventions coding manual

The coding manual for the therapist’s interventions was newly developed based on interventions of the MBT Adherence and Competency Scale (Bateman, 2018) and MBT manuals (Bateman and Fonagy, 2016; Taubner et al., 2019) by three clinical experts (LK, SH, ST). LK and SH have been trained in MBT by AFC certified MBT trainers. This deductive approach was complimented by an inductive approach in order to achieve an exhaustive coding of each statement made by the therapist. The MBT interventions coding manual (Kasper et al., 2023) (see Supplementary material S1 for definitions and examples of the interventions) includes 20 interventions. The coding of each therapist statement was performed after training (LS). It was allowed to code more than one intervention per statement. In case of ambiguity, decisions were always made in consensus. For three of the fifteen (20%) sessions, a second rating was performed. For testing inter-rater reliability with Cohen’s Kappa the multiple interventions per statement were translated into agreement or disagreement. The inter-rater reliability for the intervention coding was based on the 20 individual interventions per statement and was substantial at kappa 0.61 (Landis and Koch, 1977).

**Figure 1:**
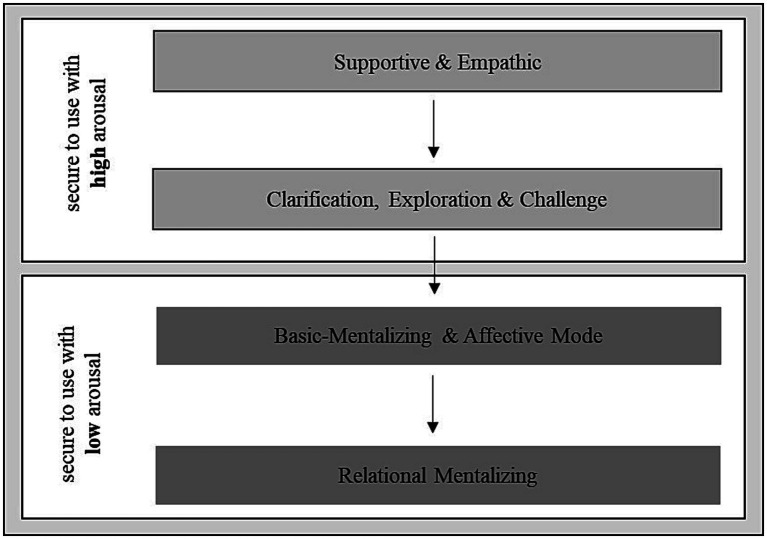
Categories of intervention levels according to the MBT-manual by Bateman and Fonagy (2016). Adapted from Taubner et al. (2019).

The interventions were categorized according to the MBT manual (Bateman and Fonagy, 2016) by three clinical experts (LK, SH, ST). The MBT manual (Bateman and Fonagy, 2016) describes a hierarchical structure of interventions, which is related to patients’ general emotional distress (arousal) (Figure 1).

When the arousal level is high, *supportive-empathic* interventions should be used to make the patient feel safe and comfortable. Interventions such as *clarification, exploration and challenge* can be used to encourage thinking about mental states. Only with a low arousal level, interventions aiming to encourage *basic mentalization* are recommended, in which affects and interpersonal experiences are explored. Furthermore, *relational mentalizing* can be used to directly address the interaction between therapist and patient.

16 interventions were summarized into the four intervention levels “supportive & empathic,” “clarification, exploration, challenge,” “basic mentalizing & affect mode” and “relational mentalizing” according to Bateman and Fonagy (2016) (Table 2). Four Interventions such as small talk, nonverbal, organizational and not classified (only 0.9%) were summarized into a fifth category “basic communication.” When the multiple intervention codings per statement differed in their corresponding intervention level, the intervention level of lower security with high arousal was chosen (in descending order: “relational mentalizing,” “basic mentalizing & affect mode,” “clarification, exploration, challenge,” “supportive & empathic”). The multiple intervention codings rarely differed in their corresponding intervention level.

**Table 2:** Interventions divided in intervention levels according to the MBT-manual (Bateman and Fonagy, 2016).

Intervention levels	Interventions
Supportive & empathic	Empathic validation Affirmation In general Regarding mentalization Self-revelation Psychoeducation/Advice Consent/Note
Clarification, exploration & challenge	Clarification (fact oriented) Stop/Stand/Rewind Change of subject Challenge Paraphrase/Interpretation Demand questions (internal cognitive processes)
Basic-mentalizing & affect mode	Offering a new perspective Mentalizing for the patient Affect-elaboration Demand Offer
Relational mentalizing	Therapist-patient-relation Affect focus

### Data analysis

Data were analyzed using the statistical program SPSS (IBM, version 28). Frequencies and percentage frequencies as well as mean values of the RF scores per patient in overall and per session were calculated. Percentage frequencies of intervention levels and interventions were calculated per patient across all sessions as well as in the high-RF sequences. RF scores and their distribution were analyzed descriptively.

To address the first research question, which intervention levels (supportive & empathic; clarification, exploration & challenge; basic-mentalizing & affect mode; relational mentalizing) are used in MBT-CD over the course of therapy, the intervention levels’ distributions were analyzed descriptively.

To address the second research question, which specific interventions and intervention levels are related to enhance effective mentalizing throughout the therapeutic process in MBT-CD, Fisher’s exact tests were used due to the large number of interventions to be tested and the low frequencies of the respective interventions in connection with effective mentalizing. Fisher’s exact tests were performed to test the differences in frequencies of interventions as well as intervention levels between high-RF sequences (RF score ≥ 4) and remaining therapy sequences (RF score ≤ 3). Because of cell frequencies less than 5, the *p* value was estimated with Fisher’s exact test using Monte Carlo simulation. One Fisher’s exact test was performed for each of the interventions and intervention levels per patient and standardized residuals z were calculated. The residuals indicate if interventions occur more frequently or less frequently than statistically expected in the sequences with high or low RF. Using the standardized residuals, conclusions can be drawn about which intervention or intervention level contributed to the potential association (Field, 2013). Effect sizes were calculated using Cramer’s V and assessed according to Cohen (1988) for Fisher’s exact tests with degrees of freedom equal to 2: a value of Cramer’s V within the range of 0.07–0.21 indicates a small effect, a value within the range of 0.21–0.35 a medium effect, and a value larger than 0.35 a large effect.

## Results

### Patients’ diagnosis and general outcomes post treatment

Thomas met criteria for ADHD, Steven met criteria for Obsessive-Compulsive Disorder and Noah did no longer fulfill criteria of Borderline and Antisocial Personality Disorder (ASPD) by the end of therapy. Regarding the GAF, all three adolescents improved at the end of therapy with 85 for Thomas, 60 for Steven and 81 for Noah.

In terms of mentalization level measured with the BRFI (Rudden et al., 2005), Thomas increased to an RF score of 3 (low mentalizing), Steven showed no improvement between surveys with a constant RF score of 3 (low mentalizing) and Noah showed no improvement with a constant RF score of 5 (ordinary mentalizing).

### Reflective functioning in session

**Figure 2:**
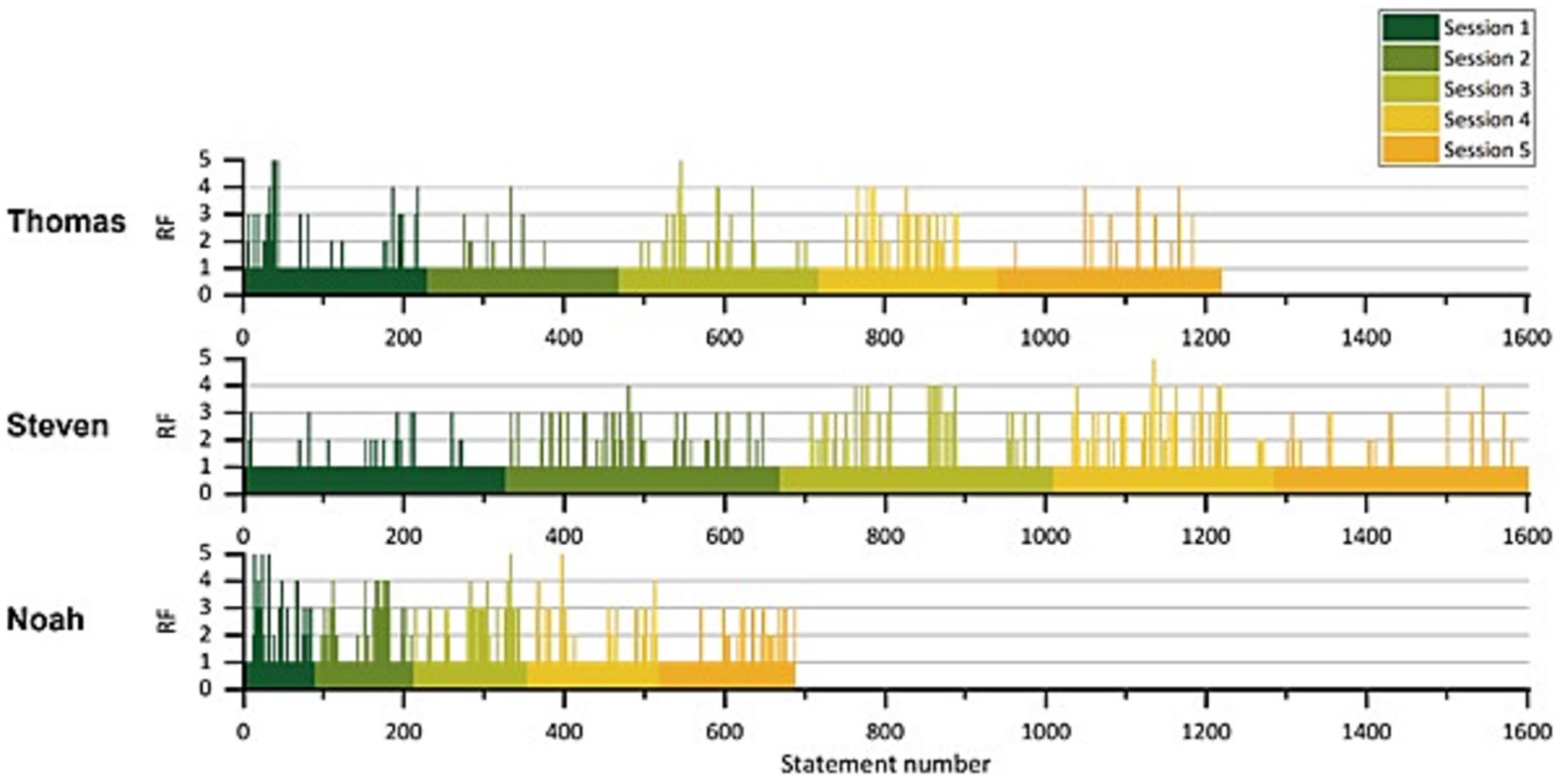
Reflective functioning per patient over the course of therapy.

In total, 3,506 patients’ statements were coded with the RF-Scale (Fonagy et al., 1998). Overall, RF in-session scores ranged from 1 to 5. Negative mentalizing (RF = −1) and above average mentalizing (RF > 5) did not occur within the selected sessions. Analyzing the course of RF scores descriptively within the sessions and across the sessions per patient (Figure 2), fluctuations can be traced. There were noticeable differences in the numbers of statements per patient: Thomas having 1,218 statements, Steven 1,602 statements and Noah 686 statements.

Thomas and Steven showed an average RF score of 1.2 and Noah an average RF score of 1.40. As the majority of patients’ statements (87.9%) were rated with an RF score of 1 (absent mentalizing). Thomas and Steven showed a higher RF value than 1 in 9.1 and 10.1% of their statements, whereas Noah mentalized twice as often with an RF score higher than 1 (22.4%).

#### High-RF

High-RF scores (ordinary mentalizing) made up a total of 1.9% of all adolescents’ statements. Thomas showed in 2% of all his statements high-RF scores, Steven in 1.3% and Noah in 3.2%.

### Distribution of the intervention levels

**Figure 3:**
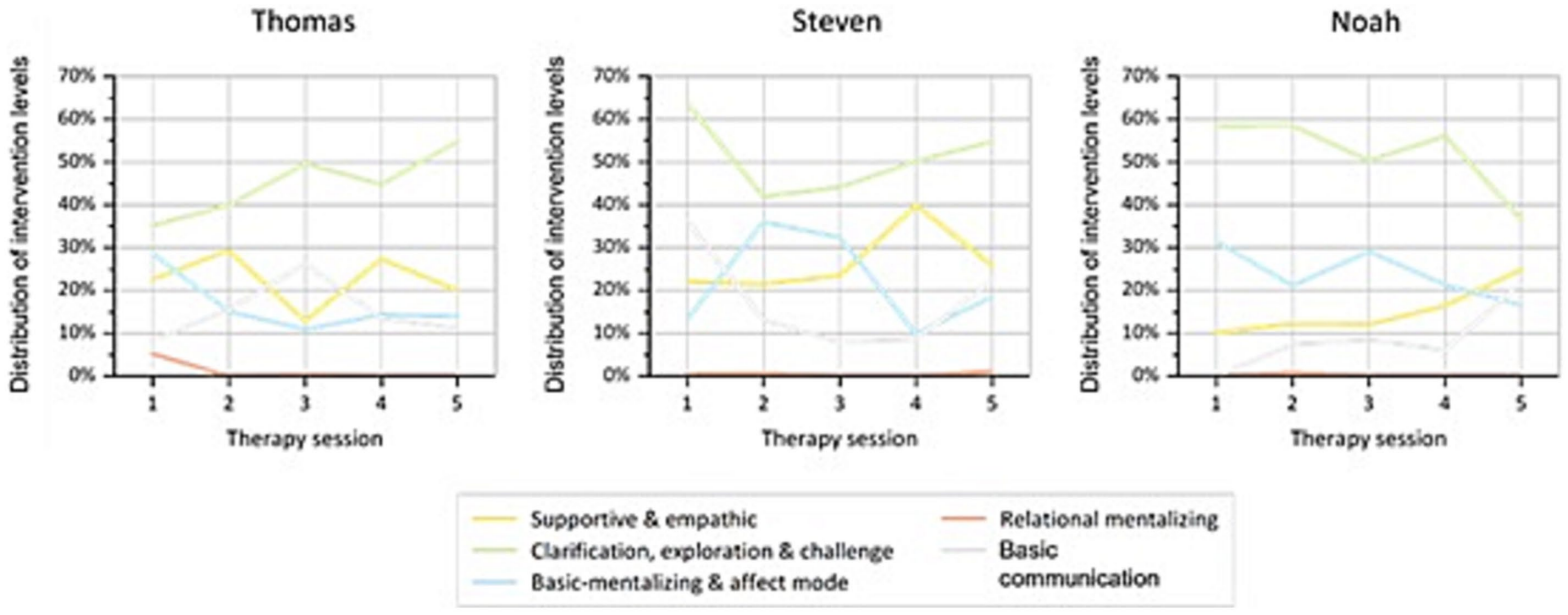
Distribution of the intervention categories used in each therapy session per patient.

Descriptive analysis of the percentage frequency of the four intervention levels and the category basic communication per patient per session over the course of therapy (Figure 3) showed that the distribution of intervention levels differed per patient between sessions but also between patients. Across all patients clarification, exploration & challenge was used most frequently (Thomas 35.2 to 54.8%; Steven 37.0 to 46.2%; Noah 36.7 to 58.5%) and relational mentalizing was used least frequently (Thomas 0 to 5.2%; Steven 0 to 1.5%; Noah 0 to 0.8%). There was no clear trend in the frequency of supportive & empathic (Thomas 12.9 to 29.4%; Steven 16.2 to 36.7%; Noah 10.1 to 24.9%) and basic-mentalizing & affect mode (Thomas 10.9 to 28.7%; Steven 9.1 to 31.8%; Noah 16.6 to 31.5%) and basic communication (Thomas 8.3 to 26.2%; Steven 7.5 to 26.6%; Noah 0 to 21.9%) across patients and course of therapy.

**Figure 4:**
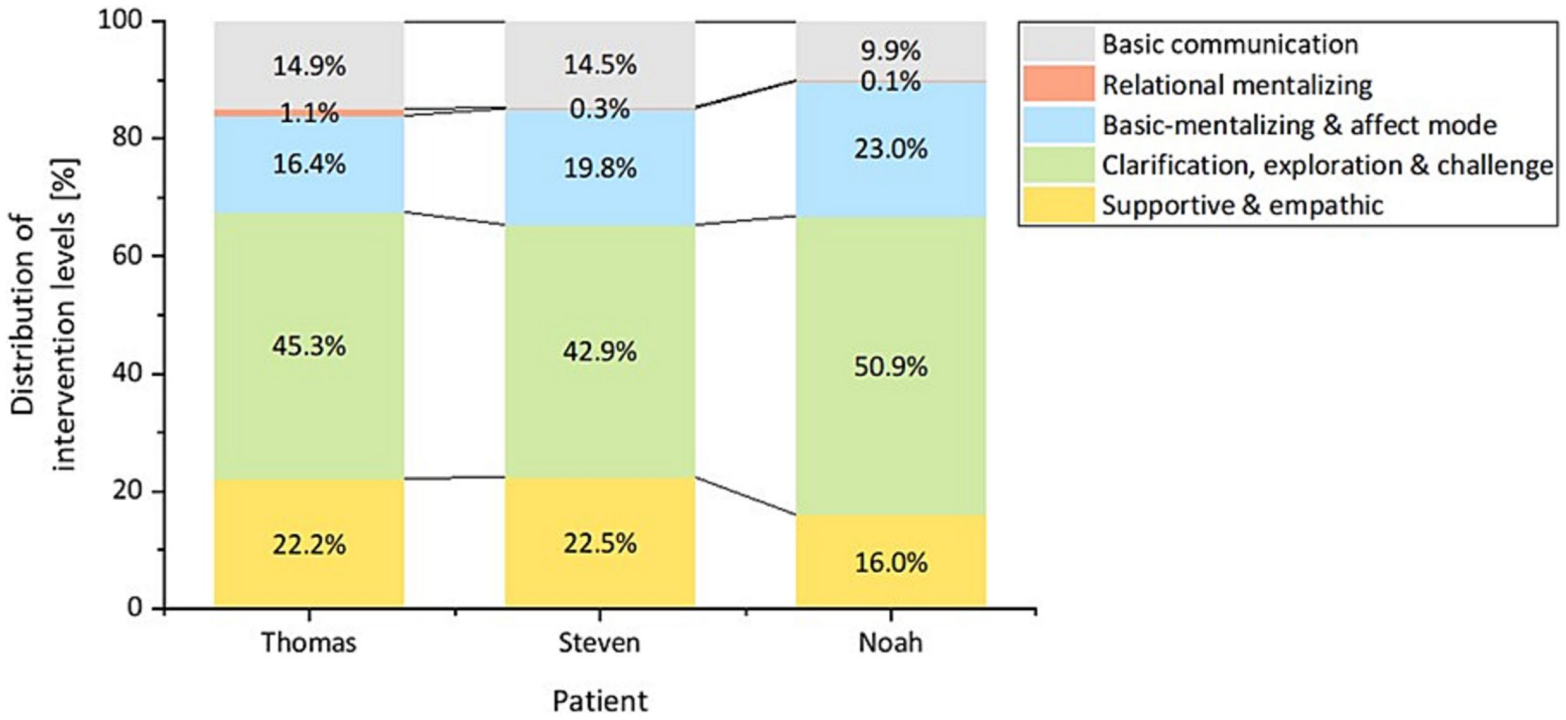
Distribution of the overall used intervention levels per patient.

The intervention levels’ distribution combined across all five therapy sessions was similar for each patient (Figure 4). Across all patients and sessions, the percentage frequency of supportive & empathic was 16.0 to 22.5%, clarification, exploration & challenge was 42.9 to 50.9%, basic-mentalizing & affect mode was 16.4 to 23.0%, relational mentalizing was 0.1 to 1.1%, and basic communication was 9.9 to 14.9%. The ranking of the intervention levels’ use was similar between patients.

### Interventions and intervention levels in high-RF sequences

In 40 cases, patients’ high-RF statements were directly preceded by the therapist’s associated content intervention. In 27 cases, it took 2 to 7 therapeutic interventions for the patient to show a content related high-RF score. 4.2% of therapeutic interventions across all sessions and patients (*n* = 153) were connected to the high-RF sequences.

The patients were analyzed as individual cases regarding their intervention and intervention level frequency differences within high-RF sequences. For each patient, a Fisher’s exact test was performed for the interventions and the intervention levels within high-RF sequences (≥ 4) in comparison to sections with low-RF values (< 4). All three adolescents showed a statistically significant association between certain interventions or intervention levels and high-RF sequences.

#### Interventions

Investigating interventions within high-RF sequences (≥ 4) and sections with low-RF values (< 4), for Thomas a large effect was evident (*V* = 0.41), for Steven a small effect (*V* = 0.19) and for Noah a medium effect (*V* = 0.28).

In Table 3 interventions associated with high-RF sequences per patient using the standardized residual z are illustrated. For Thomas, a significant association with high-RF sequences was shown for the interventions change of subject (z = 2.4, *p* < 0.05), affect-elaboration (demand) (z = 5.6, *p* < 0.001), and patient-therapist relationship (z = 12.0, *p* < 0.001). For Steven, the interventions mentalizing for the patient (z = 2.5, *p* < 0.05), empathic validation (z = 4.1, *p* < 0.001), and affect-elaboration (demand) (z = 3.7, *p* < 0.001) became significant. For Noah, demand mentalizing (z = 3.7, *p* < 0.001), challenge (z = 3.1, *p* < 0.01), and affect-elaboration (offer) (z = 2.9, *p* < 0.01) were significantly related to the high-RF sequences. A negative correlation was found with the intervention small-talk for Steven (z = 2.0, *p* < 0.05) as well as clarification for Steven (z = −2.4, *p* < 0.05) and Noah (z = −2.3, *p* < 0.05).

**Table 3:** Interventions with at least one significant association with high-RF per patient across the five sessions.

Interventions		Clarification	Demand mentalizing	Mentalizing for the patient	Empathic validation	Change of subject	Challenge	Affect-elaboration (demand)	Affect-elaboration (offer)	Patient-therapist relationship	Small-talk
Stand. Residuum *z*	Steven	**−2.4**	−0.7	**2.5**	**4.1**	−0.8	−0.6	**3.7**	−0.4	-	**−2.0**
	Thomas	−1.8	1.4	1.7	−0.9	**2.4**	−0.5	**5.6**	0.9	**12.0**	−1.4
	Noah	**−2.3**	**3.7**	1.4	0.2	−0.6	**3.1**	1.5	**2.9**	-	−1.9

Standardized residual *z* > |1.96| is significant at *p* < 0.05, z > |2.58| at *p* < 0.01, and z > |3.29| at *p* < 0.001. Significant results have been highlighted.

Table 4 shows examples of the interventions significantly positively associated with effective mentalizing.

**Table 4:** Examples of interventions successfully used to enhance the patients’ effective mentalization.

Interventions	Examples
Demand questions	Therapist: *“Ok, do you have any idea why you pushed him, although you knew that you could not allow yourself to do anything- regarding the risk of being expelled from school?“*
Affect-elaboration	Therapist: *“And then so you have a feeling about it? Can you describe that?* “(demand phrasing) Therapist: *“But I think between this “I am insecure with you “and “I need security very urgently “- in between there is somehow anger or resentment or something? “*(offering phrasing)
Empathic validation	Therapist: *“I understand, but so okay. I really understand the despair. I also understand the anger and I understand your effort not to freak out – to regulate yourself, to control yourself.“*
Challenge	Patient: *“It seems that way to me. I suppress it immediately. So when I have pain, I just make it go away inside me. They just go away like that.“* Therapist: *“How do you do that, please? Can you teach me to do that?” (laughs)*
Change of subject	Therapist: *“So Christmas was boring, okay. How was it in the family? How is it in the family at all?”*
Patient-therapist-relation	Therapist: *“But then – what just happened here between us – this pattern repeats itself inside you?“*
Mentalizing for the patient	Therapist: *“So they invest in you and then you also feel somehow valuable and think to yourself, yes, and now I‘ll really join in. Maybe that was the idea?“*

#### Intervention levels

Investigating intervention levels within high-RF sequences (≥ 4) and sections with low-RF values (< 4), for Thomas a medium effect was evident (*V* = 0.26), for Steven a small effect (*V* = 0.13) and for Noah a small effect (*V* = 0.14).

In Table 5 intervention levels associated with high-RF sequences per patient using the standardized residual z are illustrated. For Thomas, a significant association of the high-RF sequences was shown with relational mentalizing (z = 8.3, *p* < 0.001), for Steven with supportive & empathic (z = 3.4, *p* < 0.001), and for Noah with basic-mentalizing & affect mode (z = 2.6, *p* < 0.01). A negative correlation was found with the intervention level clarification, exploration & challenge (z = −2.3, *p* < 0.05) and basic communication (z = −2.1, *p* < 0.05) for Steven.

**Table 5:** Intervention levels with at least one significant association with high-RF per patient across the five sessions.

Intervention levels		Supportive & empathic	Clarification, exploration & challenge	Basic-mentalizing & affect mode	Relational mentalizing	Basic communication
Stand. Residuum *z*	Steven	**3.4**	**−2.3**	1.6	−0.4	**−2.1**
	Thomas	−1.4	−0.2	1.6	**8.3**	−1.7
	Noah	−1.7	−0.1	**2.6**	−0.2	−1.5

Standardized residual *z* > |1.96| is significant at *p* < 0.05, z > |2.58| at *p* < 0.01, and *z* > |3.29| at *p* < 0.001. Significant results have been highlighted.

## Discussion

The goal of the multiple MBT-CD case study was an exploratory investigation of therapeutic interventions categorized to intervention levels regarding a general use and their percentage frequencies over the course of therapy. Furthermore, the study aimed to analyze the influence of specific interventions on effective mentalizing. Over the course of therapy, a similarly frequent use of intervention levels between patients was shown. Across all patients and sessions the most frequently used intervention level was clarification, exploration & challenge and the least frequently used was relational mentalizing. This was also evident in the patients’ individual sessions, although there were differences between therapy sessions per patient and between patients. There was no clear upward or downward tendency visible in the intervention levels’ frequencies of use across the course of therapy. A variety of interventions, such as demand questions, affect-elaboration, empathic validation, challenge, change of subject, patient-therapist relation and mentalizing for the patient, were successfully used to enhance the patients’ effective mentalization. Regarding intervention levels, supportive & empathic, basic- mentalizing & affect mode and relational mentalizing were positively related to effective mentalizing. Interventions and intervention levels enhancing effective mentalizing seem to be patient-specific and might depend on various variables, such as the patients’ arousal and pre-mentalizing mode or therapists’ mentalizing.

In order to analyze interventions in MBT-CD and their specific relation to effective mentalizing, three patients were selected. The patients are characteristic for MBT-CD target group in relation to their symptoms and show a good treatment outcome regarding their diagnosis. However, two of three patients did not show an improvement in their mentalization, measured with the BRFI (Rudden et al., 2005), at the end of therapy. Only Thomas mentalizing level, being the lowest mentalizing level of all patients (absent to lacking) at baseline, was increased to the low level at the end of therapy. Thomas (RF_pre_ = 2, RF_post_ = 3) and Steven (RF = 2) correspond to the average low mentalizing level (RF = 2.6) of violent adolescents (Taubner et al., 2010) showing a partial understanding of intentions of self and others. Noah stood out with an ordinary mentalizing level (RF = 5) showing a consistent model for thoughts and feelings of self and others, which is above the average mentalizing level of healthy adolescents (RF = 3.17–4.7) (Taubner et al., 2013; Borelli et al., 2015; Cropp et al., 2019a). Overall, it should be emphasized that only Thomas showed an improvement in mentalization compared to before and after therapy. Nevertheless, there was an improvement in the diagnosis for all three adolescents, so the relationship between mentalization and treatment outcome should be investigated in further studies in more detail.

Aiming to understand the mechanisms of MBT the study furthermore focused on mentalizing as a state during therapy sessions. Overall, there was an above-average frequency of absent mentalizing. Within therapy sessions, all three adolescents showed similar lacking to low mentalizing, mentioning mental states with some evidence of consideration of mental states without explicitness. Noah mentalized twice as often as the other two patients, whereby he used mental states to explain behavior in an accurate way. It should be noted that every statement made by patients, and not just statements related to demand questions, were rated using the RF Scale (Talia et al., 2015). The assessment of monosyllabic responses or the mere absence of mentalizing when not prompted by the therapist may have led to an underestimation of patients’ mentalizing ability. However, to examine the relationship between each possible therapist intervention and patient mentalizing, it was necessary to assess each patient statement. Within sessions and across sessions fluctuations of mentalizing were visible as in other studies (Hörz-Sagstetter et al., 2015; Kornhas et al., 2020; Kivity et al., 2021). This underlines the hypotheses that mentalizing rather is a state than a trait during therapy sessions. It can be assumed that it is not in general about a mentalizing increase within therapy sessions or over the course of therapy, but about the production of moments in which mentalizing is increased and thus consequently an inner change of mental states takes place when effective mentalizing is generated. More precisely, it is about strengthening the mentalization of certain aspects of life that could not be mentalized before, such as certain triggers (When do they become violent? What are the catalysts?).

### Use of intervention levels

To examine if specific therapeutic interventions are used in MBT-CD, an exhaustive rating instrument was developed. Subsequently, the 22 interventions of the coding instrument were transformed into Bateman and Fonagy’s (2016) four-level model, requiring the incorporation of basic communication as an additional category including interventions such as non-verbal response, small talk, and session organization. Based on the MBT manual (Bateman and Fonagy, 2016), the use of intervention levels is adjusted to the patient’s arousal state following a hierarchical order. Accordingly, if arousal is high, the following intervention levels should be used in the following order: supportive & empathic; clarification, exploration & challenge. If arousal is low, the following intervention levels should be used in the following order: basic-mentalizing & affect mode; relational mentalizing. According to the MBT manual (Bateman and Fonagy, 2016), relational mentalizing should only be used, when the patient is in a state of effective mentalizing. In the current study, the patients’ percentage frequencies of intervention levels were similar across the sessions, although the percentage frequencies of intervention levels per session differed between and within patients. Across all patients and sessions, the most frequently used intervention level was clarification, exploration & challenge and the least frequently used intervention level was relational mentalizing. This pattern of use was also evident in the patients’ individual sessions. There was no clear progression in the intervention levels’ frequencies. Since the intervention levels supportive & empathic as well as clarification, exploration & challenge were frequently used, it can be assumed that high arousal was present in the patients at the time of use. This conclusion is consistent with the theoretical background of the intervention levels (Bateman and Fonagy, 2016). To test this assumption, arousal should be included in future studies.

Overall, all theoretically anticipated levels of the model (Bateman and Fonagy, 2016) were used within the therapy sessions. However, use of the relational mentalizing level was conspicuously low (0.1–1.1%), although relational mentalizing is known to be a core element of MBT. This result is similar to the findings of a study of a mentalization based long-term treatment for an adult patient with borderline personality disorder, which was carried out by the same study therapist (ST) (Kornhas et al., 2020). In addition, previous studies of adult populations have also found that this intervention despite its importance is not very frequently used compared to other MBT interventions (Karterud et al., 2013; Simonsen et al., 2018). Over the course of this treatment, the patient-therapist relationship was rarely a subject of conversation. On the one hand, these results might be explained by the patients arousal being too high or the patients mentalizing being too low. On the other hand, the therapist, despite a general adherence to the MBT manual (Bateman and Fonagy, 2016), might have hardly used relational mentalizing as an intervention. This hypothesis is supported by a study by Karterud et al. (2013), whereas the low use of patient-therapist intervention appeared to be therapist-specific. If this is the case, special therapist training for this kind of intervention might be needed.

### Effective mentalizing

Of particular interest for understanding mentalizing processes and their mechanisms of change are sequences of high mentalizing. In these moments, a patient’s mentalizing space expands and effective mentalizing possibly occurs (Allen et al., 2008). Accordingly, moments with above-average mentalizing resemble an effective mentalizing experience. In order to obtain a better understanding of interventions related to effective mentalizing, a sequence of content-related interventions prior to high mentalizing was formed and analyzed. High mentalizing was positively related to the intervention affect-elaboration in all three cases, whereby Steven and Thomas benefitted from a demanding and Noah from an offering phrasing. However, Noah significantly responded to demand questions aiming at cognitive internal processes with high mentalizing. It can therefore be concluded that for all three adolescents demand questions play an important positive role regarding high mentalizing, either related to cognitive or affective internal processes. This supports the assumption that demand questions are an important intervention within MBT in theory and practice and increase mentalizing (Möller et al., 2017; Kornhas et al., 2020; Kivity et al., 2021).

Furthermore, in the current study, other interventions aside demand questions were related to high mentalizing and therefore support results from Meier et al. (2023). Specific interventions such as affect-elaboration (offering), challenge, change of subject, patient-therapist relation, empathic validation and mentalizing for the patient were associated with high mentalizing. Thus, it can be postulated that MBT interventions can indeed promote effective mentalizing at various intervention levels. Although intervention levels were used similarly frequent per patient over the course of therapy, individual differences regarding effective mentalizing-promoting interventions can be identified. It can be assumed that the patient-specific use of the interventions prior to effective mentalizing is related to the patients’ arousal level and pre-mentalizing mode. Depending on the patient’s pre-mentalizing mode, specific intervention are suggested to establish mentalizing (Bateman and Fonagy, 2016). Interventions which were positively associated to high mentalizing, such as change of subject, challenge and empathic validation, belong to interventions challenging pre-mentalizing. Another intervention positively associated with high mentalizing was mentalizing for the patient. While the MBT manual advises not to mentalize for the patients, but rather use questions and statements to encourage the patient to mentalize (Bateman and Fonagy, 2016), mentalizing for the patients has been related to improving mentalizing before (Georg et al., 2019). Mentalizing for the patient can entail the risk that the patient pseudo-mentalizes, i.e., goes along with what the therapist says and reflects mental states without emotional coherence. On the other hand, the therapist’s ability to draw out subdominant aspects of the patient’s narrative is increasingly seen as part of establishing a sense of ‘we’ and epistemic trust within the therapy. The patient-therapist relation was significantly positively associated with high mentalizing. This intervention was hardly used, but if so, it had a great effect on mentalizing. Knowing that this intervention is only used when the patient is in a mentalizing state (Bateman and Fonagy, 2016) and considering its high effect on mentalizing, the question is raised, whether working on the therapeutic relationship would also be effective when the patient is not in a mentalizing state. It might be beneficial to challenge the patients’ comfort zone with a clear therapeutic stance to foster the therapeutic process as seen in a study on patients with borderline personality disorder (Folmo et al., 2019). Supporting this approach, it has been shown in a dismantling study of depressive adolescents that the exploration of the adolescents’ relations to the therapist amplified the effects of short-term psychoanalytic psychotherapy on their depressive symptoms (Ullberg et al., 2015). In addition, interventions with regards to the patient-therapist relation seemed to be especially important for patients with long-standing, more severe interpersonal problems in a dismantling study of one year psychodynamic adult-therapies (Hoglend et al., 2008). Furthermore, three out of four levels of the manual based model (Bateman and Fonagy, 2016) were significantly positively related to higher mentalizing in a patient-specific manner: supportive & empathic; basic- mentalizing & affect mode; relational mentalizing. Concluding, effective mentalizing can occur through each of these three MBT intervention levels in Bateman and Fonagy (2016) adolescents with CD.

In contrast, clarification, exploration & challenge and basic communication as well as the interventions clarification and small-talk were significantly negatively associated with high mentalizing for two patients. This can be caused by the fact that clarification focusses on facts to get a better understanding of the patients’ narratives, whereas this definition of clarification is not consistent with the MBT manual (Bateman and Fonagy, 2016). Therefore, it can be assumed that a lot of non-mentalizing report about scenes, etc. is included in clarification. It could also be that clarification might be signaling epistemic distrust of the patient (i.e., “what you say is not making much sense and I need to know more in order for me trust you”). This is particularly important for the patient group with CD, as they are particularly vulnerable in terms of not being believed and trusted (Talia et al., 2021). The definition of clarification should be specified more clearly in further studies.

However, non-significant or negative significant relations do not allow to assess whether these interventions or intervention levels are in general ineffective to increase mentalizing, since the causal dependence is not known. Furthermore, it is conceivable that a variety of interventions, which are not associated with high mentalizing, are important for understanding what is being said or for building a sustainable relationship. Particularly for young adults with higher arousal, the intervention small talk could be anxiety-reducing and pave the way for a trusting relationship.

Moreover, there are some additional factors that could contribute to the interaction between therapeutic interventions and effective mentalizing. It is of interest to examine whether different interventions or intervention levels are used depending on the patient’s arousal level. This could also test the hypothesis of the MBT manual (Bateman and Fonagy, 2016) according to which intervention levels are used depending on the patient’s arousal. Inspired by Kivity et al. (2021) it is of interest to analyze the interaction of patients’ arousal, mentalizing and a range of therapeutic interventions. To capture arousal, patients’ talking turns could be acoustically encoded (Kivity et al., 2021). In addition, it seems to be important to include the mentalizing level of the therapist in future investigations because the patients’ mentalizing was found to increase when the therapist uses a similar mentalizing level compared to that of the patients (Diamond et al., 2003; de la Cerda and Dagnino, 2021). Therefore, it might be beneficial to tailor interventions to the patient’s level of mentalizing (Diamond et al., 2003; Kasper et al., in prep)^2^. Two other important points within MBT are to counterbalance imbalances of patients’ mentalization dimensions (implicit/explicit, self/other, cognitive/affective, internal/external) and to contrast patients’ pre-mentalizing modes. It can be assumed that these also influence the choice of interventions and thus an increase in effective mentalizing.

### Limitations

Results obtained in the exploratory, multiple case study should be regarded as promising initial indications for further research and theory building. However, number of patients and selected therapy sessions are to be named as a limitation, since no generalizable statements can be made thereby. Furthermore, three therapies of only one therapist were used to minimize therapist variance. However, despite recognition as an MBT therapist and proven adherence to the sessions, individual characteristics in the implementation of MBT may play a role.

Overall, the MBT interventions coding manual (Kasper et al., 2023) was newly developed and requires further validation. As part of the exploratory approach, the coded interventions were assigned to Bateman and Fonagy’s (2016) model by consensus of three raters. In particular, the very high number of the intervention clarification may have had an impact on the evaluation of the intervention level clarification, exploration & challenge and is not consistent with the original definition of clarification by Bateman and Fonagy (2016). Furthermore, interventions can often be interrelated. Therefore, it is artificial to build high mentalizing sequences in order to analyze the interventions that enhance mentalization. It could also be that even the best mentalizing intervention will not lead to improved RF in the context of a poor therapeutic relationship. As previously discussed, other factors seem to play an important role that were not included in the study like patients’ pre-mentalizing modes and arousal level as well as therapist mentalizing and quality of the patient-therapist relation.

### Research and clinical implications

Identifying effective therapy components is necessary to improve therapy and corresponding manuals (Kazdin, 2003). Regarding MBT this means to better understand the mentalizing process. For this purpose, a uniform approach to mentalizing assessment is desirable to ensure comparability between studies which analyze in-session mentalizing processes. Previous studies have used different ways to capture mentalizing within therapy sessions using the RF scale, whereby the patients’ talking unit varied greatly (Talia et al., 2019). To examine a patients’ mentalizing level in response to prior interventions, the approach of coding statement-by-statement (Möller et al., 2017; Kivity et al., 2021) was proven appropriate. However, the strong differences in statement numbers between patients were striking. The unequal statement numbers and their meaning in therapy should be considered in more detail in further research. The methodological implementation of using high mentalizing scores and forming sequences represents a promising approach for investigating interventions related to effective mentalizing.

Overall, interventions used in therapy should be investigated in more detail, focusing on interventions positively related to effective mentalizing besides demand questions: Firstly, affect-elaboration with an offering phrasing; secondly, relational mentalizing, because of its rare use despite its importance within MBT; thirdly, mentalizing for the patient to clarify its role within MBT; fourthly, interventions potentially related to pre-mentalizing modes, such as change of subject, challenge and empathic validation. Future studies should aim at dismantling interventions by controlling their use like it has been demonstrated for transference interpretations (Ullberg et al., 2015). In general, the additional factors pre-mentalizing mode, patients’ arousal, and therapists’ mentalizing level are recommended to be considered in further investigations of interventions within MBT. This aligns with the recommendations of Meier et al. (2023), who also highlight the importance of additional factors, including patient-therapist relation.

Specific MBT interventions can be associated with effective mentalizing. Clinically, the most promising interventions might be demand questions, affect-elaboration (offering and demanding phrasing), change of subject, challenge, patient-therapist relation, empathic validation and mentalizing for the patient. However, interventions that promote high mentalizing sequences may differ between patients. Accordingly, therapists need to tailor interventions individually to identify those interventions that promote effective mentalizing.

## Data availability statement

The datasets presented in this article are not readily available because as this is sensitive patient information no data can be disclosed. Requests to access the datasets should be directed to Lea.Kasper@med.uni-heidelberg.de.

## Ethics statement

The studies involving humans were approved by the Ethics Committee of the Heidelberg University Medical Faculty (Germany). The studies were conducted in accordance with the local legislation and institutional requirements. Written informed consent for participation in this study was provided by the participants and their legal guardians.

## Author contributions

LK: conceptualization, writing – original draft, and project administration. LK, SH, and ST: methodology and writing – review & editing. ST: resources and supervision. LK and LS: formal analysis, investigation, data curation, and visualization. All authors have read and agreed to the published version of the manuscript.

## Footnotes

^1^Names have been changed.

^2^Kasper, L. A., Krivzov, J., Diederich J. and Taubner, S. (in prep.). Changes of Mentalization during Psychotherapy - a Metasynthesis.

## References

[ref1] Allen J. G. Fonagy P. Bateman A. W. (2008). Mentalizing in clinical practice. Washington, DC: American Psychiatric Pub.

[ref2] American Psychiatric Association (2013). Diagnostic and statistical manual of mental disorders (DSM-5®). Washington, DC: American Psychiatric Pub.

[ref3] Babl A. Berger T. Decurtins H. Gross I. Frey T. Caspar F.. (2022). A longitudinal analysis of reflective functioning and its association with psychotherapy outcome in patients with depressive and anxiety disorders. J. Couns. Psychol. 69, 337–347. doi: 10.1037/cou0000587 34618487

[ref4] Bateman A. W. (2018). Mentalization-based treatment: Adherence and competence scale. London: Anna Freud National Centre for Children and Families.

[ref5] Bateman A. Fonagy P. (2016). Mentalization based treatment for personality disorders: A practical guide. Oxford: Oxford University Press.

[ref6] Borelli J. L. Compare A. Snavely J. E. Decio V. (2015). Reflective functioning moderates the association between perceptions of parental neglect and attachment in adolescence. Psychoanal. Psychol. 32, 23–35. doi: 10.1037/a0037858

[ref7] Cohen J. (1988). Statistical power analysis for the behavioral sciences. Abingdon. London: Routledge.

[ref8] Costello E. J. Mustillo S. Erkanli A. Keeler G. Angold A. (2003). Prevalence and development of psychiatric disorders in childhood and adolescence. Arch. Gen. Psychiatry 60, 837–844. doi: 10.1001/archpsyc.60.8.837 12912767

[ref9] Cropp C. Alexandrowicz R. W. Taubner S. (2019a). Reflective functioning in an adolescent community sample. Mental Health & Prevention 14:200156. doi: 10.1016/j.mph.2019.200156

[ref10] Cropp C. Taubner S. Salzer S. Streeck-Fischer A. (2019b). Psychodynamic psychotherapy with severely disturbed adolescents: changes in reflective functioning. J. Infant Child Adolescent Psychother. 18, 263–273. doi: 10.1080/15289168.2019.1643212

[ref11] de la Cerda C. Dagnino P. (2021). In-session reflective functioning: relationship with the presence and depth of work on conflict or personality functioning. Front. Psychol. 12, 39–48. doi: 10.3389/fpsyg.2021.725739 PMC8488170 34616342

[ref12] Diamond D. Clarkin J. F. Chase Stovall-McClough K. Levy K. N. Foelsch P. A. Levine H.. (2003). Patient-therapist attachment: Impact on the therapeutic process and outcome. London: Whurr Publishers.

[ref13] Ekeblad A. Falkenströn F. Vestberg R. Holmqvist R. (2016). Randomized trial of interpersonal psychotherapy and cognitive behavioral therpay for major depressive disorder in a community-based psychiatric outpatienr clinic. Depress. Anxiety 33, 1090–1098. doi: 10.1002/da.22495, PMID: 27029912

[ref14] Field A. (2013). Discovering statistics using IBM SPSS statistics. Thousand Oaks, CA: Sage.

[ref15] Folmo E. J. Karterud S. W. Kongerslev M. T. Kvarstein E. H. Stänicke E. (2019). Battles of the comfort zone: modelling therapeutic strategy, Alliance, and epistemic trust—a qualitative study of Mentalization-based therapy for borderline personality disorder. J. Contemp. Psychother. 49, 141–151. doi: 10.1007/s10879-018-09414-3

[ref16] Fonagy P. Gergely G. Jurist E. Target M. (2002). Affect regulation, mentalization, and the development of the self. New York: Other Press.

[ref17] Fonagy P. Target M. Steele H. Steele M. (1998). Reflective-functioning manual version 5 for application to adult attachment interviews. London: University College London.

[ref9001] Fydrich T. Renneberg B. Schmitz B. Wittchen H. U. (1997). Strukturiertes Klinisches interview für DSM-IV, Achse II (SKID II). [The structured clinical interview for DSM-IV, Axis-II, SCID-II]. Göttingen, Hogrefe.

[ref18] Georg A. Kress S. Taubner S. (2019). Strengthening mentalizing in a depressed mother of an infant with sleep disorders. J. Clin. Psychol. 75, 859–873. doi: 10.1002/jclp.22762, PMID: 30793312

[ref19] George C. Main M. Kaplan N. (1996). Adult attachment interview. Interpersona: An International Journal on Personal Relationships.

[ref9003] Hall R. C. (1995). Global assessment of functioning: a modified scale. Psychosomatics, 36, 267–275. 7638314 10.1016/S0033-3182(95)71666-8

[ref20] Hauschild S. Kasper L. A. Taubner S. (2021). “Was hat dir an der Therapie gefallen?” Eine qualitative Studie zur Evaluation der Mentalisierungsbasierten Therapie für Jugendliche mit Störungen des Sozialverhaltens. Prax. Kinderpsychol. Kinderpsychiatr. 70, 386–402. doi: 10.13109/prkk.2021.70.5.386, PMID: 34187334

[ref21] Hauschild S. Kasper L. Volkert J. Sobanski E. Taubner S.. (2023). Mentalization-based treatment for adolescents with conduct disorder (MBT-CD): a feasibility study. Eur Child Adolesc Psychiatry. 32, 2611–2622. doi: 10.1007/s00787-022-02113-4 36434148 PMC9702655

[ref22] Hoglend P. Bogwald K. P. Amlo S. Marble A. Ulberg R. Sjaastad M. C.. (2008). Transference interpretations in dynamic psychotherapy: do they really yield sustained effects? Am. J. Psychiatry 165, 763–771. doi: 10.1176/appi.ajp.2008.07061028, PMID: 18413707

[ref23] Hörz-Sagstetter S. Mertens W. Isphording S. Buchheim A. Taubner S. (2015). Changes in reflective functioning during psychoanalytic psychotherapies. J. Am. Psychoanal. Assoc. 63, 481–509. doi: 10.1177/0003065115591977, PMID: 26185290

[ref24] Karterud S. Pedersen G. Engen M. Johansen M. S. Johansson P. N. Schluter C.. (2013). The MBT adherence and competence scale (MBT-ACS): development, structure and reliability. Psychother. Res. 23, 705–717. doi: 10.1080/10503307.2012.708795, PMID: 22916991

[ref9005] Kasper L. A. Hauschild S. Schrauf L. M. Taubner S. (2023). MBT interventions coding manual. Fpsyg, 14. 10.3389/fpsyg.2023.1223040 PMC10800920 38259532

[ref25] Katznelson H. (2014). Reflective functioning: a review. Clin. Psychol. Rev. 34, 107–117. doi: 10.1016/j.cpr.2013.12.003 24486522

[ref26] Kazdin A. E. (2003). Psychotherapy for children and adolescents. Annu. Rev. Psychol. 54, 253–276. doi: 10.1146/annurev.psych.54.101601.145105 12185210

[ref27] Kivity Y. Levy K. N. Kelly K. M. Clarkin J. F. (2021). In-session reflective functioning in psychotherapies for borderline personality disorder: the emotion regulatory role of reflective functioning. J. Consult. Clin. Psychol. 89, 751–761. doi: 10.1037/ccp0000674, PMID: 34591548 PMC9634511

[ref28] Koo T. K. Li M. Y. (2016). A guideline of selecting and reporting intraclass correlation coefficients for reliability research. J. Chiropr. Med. 15, 155–163. doi: 10.1016/j.jcm.2016.02.012, PMID: 27330520 PMC4913118

[ref29] Kornhas L. A. Schröder-Pfeifer P. Georg A. Zettl M. Taubner S. (2020). Process of mentalization in a mentalization-based long-term therapy for borderline personality disorders: a case study. Psychotherapeut 65, 357–365. doi: 10.1007/s00278-020-00451-9

[ref30] Lahey B. B. Loeber R. Burke J. D. Applegate B. (2005). Predicting future antisocial personality disorder in males from a clinical assessment in childhood. J. Consult. Clin. Psychol. 73, 389–399. doi: 10.1037/0022-006X.73.3.389, PMID: 15982137

[ref31] Landis J. R. Koch G. G. (1977). The measurement of observer agreement for categorical data. Biometrics 33:159. doi: 10.2307/2529310 843571

[ref32] Lemma A. Target M. Fonagy P. (2010). The development of a brief psychodynamic protocol for depression: dynamic interpersonal therapy (DIT). Psychoanal. Psychother. 24, 329–346. doi: 10.1080/02668734.2010.513547

[ref33] Levy K. N. Meehan K. B. Kelly K. M. Reynoso J. S. Weber M. Clarkin J. F.. (2006). Change in attachment patterns and reflective function in a randomized control trial of transference-focused psychotherapy for borderline personality disorder. J. Consult. Clin. Psychol. 74, 1027–1040. doi: 10.1037/0022-006X.74.6.1027, PMID: 17154733

[ref34] Luyten P. Van Houdenhove B. Lemma A. Target M. Fonagy P. (2012). A mentalization-based approach to the understanding and treatment of functional somatic disorders. Psychoanal. Psychother. 26, 121–140. doi: 10.1080/02668734.2012.678061

[ref35] Meier A. F. Zeeck A. Taubner S. Gablonski T. Lau I. Preiter R.. (2023). Mentalization-enhancing therapeutic interventions in the psychotherapy of anorexia nervosa: An analysis of use and influence on patients’ mentalizing capacity. Psychotherapy Research, 33, 595–607. doi: 10.1080/10503307.2022.2146542 36473209

[ref36] Möller C. Falkenström F. Holmqvist Larsson M. Holmqvist R. (2014). Mentalizing in young offenders. Psychoanal. Psychol. 31, 84–99. doi: 10.1037/a0035555

[ref37] Möller C. Karlgren L. Sandell A. Falkenström F. Philips B. (2017). Mentalization-based therapy adherence and competence stimulates in-session mentalization. Psychother. Res. 27, 749–765. doi: 10.1080/10503307.2016.1158433 27093128

[ref38] Morosan L. Ghisletta P. Badoud D. Toffel E. Eliez S. Debban Ã. M. (2020). Longitudinal relationships between reflective functioning, empathy, and externalizing behaviors during adolescence and young adulthood. Child Psychiatry Hum. Dev. 51, 59–70. doi: 10.1007/s10578-019-00910-8 31309356

[ref39] Newbury-Helps J. Feigenbaum J. Fonagy P. (2017). Offenders with antisocial personality disorder display more impairments in mentalizing. J. Personal. Disord. 31, 232–255. doi: 10.1521/pedi_2016_30_246, PMID: 27064853

[ref40] Pardini D. Frick P. J. (2013). Multiple developmental pathways to conduct disorder: current conceptualizations and clinical implications. J. Can. Acad. Child Adolesc. Psychiatry 22, 20–25. PMID: 23390429 PMC3565711

[ref41] Petermann F. Petermann U. (2013). Störungen des Sozialverhaltens. Kindheit und Entwicklung 22, 123–126. doi: 10.1026/0942-5403/a000108

[ref42] Polanczyk G. V. Salum G. A. Sugaya L. S. Caye A. Rohde L. A. (2015). Annual research review: a meta-analysis of the worldwide prevalence of mental disorders in children and adolescents. J. Child Psychol. Psychiatry 56, 345–365. doi: 10.1111/jcpp.12381 25649325

[ref43] Ravens-Sieberer U. Wille N. Erhart M. Bettge S. Wittchen H.-U. Rothenberger A.. (2008). Prevalence of mental health problems among children and adolescents in Germany: results of the BELLA study within the National Health Interview and examination survey. Eur. Child Adolesc. Psychiatry 17, 22–33. doi: 10.1007/s00787-008-1003-2 19132301

[ref44] Ridenour T. A. Cottler L. B. Robins L. N. Campton W. M. Spitznagel E. L. Cunningham-Williams R. M. (2002). Test of the plausibility of adolescent substance use playing a causal role in developing adulthood antisocial behavior. J. Abnorm. Psychol. 111, 144–155. doi: 10.1037/0021-843X.111.1.144, PMID: 11866167

[ref45] Rudden M. G. Milrod B. Target M. (2005). The brief reflective functioning interview. New York: Weill Cornell Medical College.

[ref9007] Sheehan D. V. Shytle D. Milo K. Janavs J. Lecrubier Y. (2010). Mini international neuropsychiatric interview for children and adolescents (MINI-KID). J Clin Psychiatry, 71, 313–26. 20331933 10.4088/JCP.09m05305whi

[ref46] Simonsen S. Juul S. Kongerslev M. Bo S. Folmo E. Karterud S. (2018). The mentalization-based therapy adherence and quality scale (MBT-AQS): reliability in a clinical setting. Nordic Psychol. 71, 104–115. doi: 10.1080/19012276.2018.1480406

[ref47] Talia A. Duschinsky R. Mazzarella D. Hauschild S. Taubner S. (2021). Epistemic trust and the emergence of conduct problems: aggression in the Service of Communication. Front. Psych. 12:710011. doi: 10.3389/fpsyt.2021.710011, PMID: 34630177 PMC8494977

[ref48] Talia A. Miller-Bottome M. Katznelson H. Pedersen S. H. Steele H. Schröder P.. (2019). Mentalizing in the presence of another: measuring reflective functioning and attachment in the therapy process. Psychother. Res. 29, 652–665. doi: 10.1080/10503307.2017.1417651, PMID: 29298602 PMC6102086

[ref49] Talia A. Steele H. Taubner S. (2015). In-session RF coding manual. Heidelberg: University of Heidelberg.

[ref50] Taubner S. (2008a). Mentalisierung und Einsicht. Forum der Psychoanalyse 24, 16–31. doi: 10.1007/s00451-008-0340-6

[ref51] Taubner S. (2008b). Entsteht Einsicht im Täter-Opfer-Ausgleich? Eine empirische Studie am Beispiel adoleszenter Gewaltstraftäter. Monatsschrift für Kriminologie und Strafrechtsreform 91, 281–294. doi: 10.1515/mks-2008-910404

[ref52] Taubner S. Buchheim A. Kächele H. Staun L. (2011). The role of mentalization in the psychoanalytic treatment of chronic depression. Psychiatry Interpers Biol Proc 74, 49–57. doi: 10.1521/psyc.2011.74.1.49 21463170

[ref53] Taubner S. Fonagy P. Bateman A. W. (2019). Mentalisierungsbasierte Therapie. Göttingen: Hogrefe Verlag.

[ref54] Taubner S. Hauschild S. Kasper L. Kaess M. Sobanski E. Gablonski T.-C.. (2021). Mentalization-based treatment for adolescents with conduct disorder (MBT-CD): protocol of a feasibility and pilot study. Pilot Feasibil. Stud. 7, 1–10. doi: 10.1186/s40814-021-00876-2 PMC8252214 34215323

[ref55] Taubner S. Sevecke K. (2015). Core model of mentalization-based treatment. Psychotherapeut 60, 169–184. doi: 10.1007/s00278-015-0012-0

[ref56] Taubner S. White L. O. Zimmermann J. Fonagy P. Nolte T. (2013). Attachment-related mentalization moderates the relationship between psychopathic traits and proactive aggression in adolescence. J. Abnorm. Child Psychol. 41, 929–938. doi: 10.1007/s10802-013-9736-x 23512713

[ref57] Taubner S. Wiswede D. Nolte T. Roth G. (2010). Mentalisierung und externalisierende Verhaltensstörungen in der Adoleszenz. Psychotherapeut 55, 312–320. doi: 10.1007/s00278-010-0753-8

[ref58] Taubner S. Zimmermann L. Ramberg A. Schröder P. (2016). Mentalization mediates the relationship between early maltreatment and potential for violence in adolescence. Psychopathology 49, 236–246. doi: 10.1159/000448053, PMID: 27548462

[ref59] Ullberg T. Zia E. Petersson J. Norrving B. (2015). Changes in functional outcome over the first year after stroke: an observational study from the Swedish stroke register. Stroke 46, 389–394. doi: 10.1161/STROKEAHA.114.006538 25538204

[ref60] Vloet T. D. Herpertz S. Herpertz-Dahlmann B. (2006). Ätiologie und Verlauf kindlichen dissozialen Verhaltens-Risikofaktoren für die Entwicklung einer antisozialen Persönlichkeitsstörung. Zeitschrift für Kinder-und Jugendpsychiatrie und Psychotherapie 34, 101–116. doi: 10.1024/1422-4917.34.2.101, PMID: 16610596

[ref61] Volkert J. Hauschild S. Taubner S. (2019). Mentalization-based treatment for personality disorders: efficacy, effectiveness, and new developments. Curr. Psychiatry Rep. 21, 1–12. doi: 10.1007/s11920-019-1012-5 30852694

[ref62] Weijers J. Ten Kate C. Eurelings-Bontekoe E. Viechtbauer W. Rampaart R. Bateman A.. (2016). Mentalization-based treatment for psychotic disorder: protocol of a randomized controlled trial. BMC Psychiatry 16, 1–10. doi: 10.1186/s12888-016-0902-x 27278250 PMC4898403

[ref63] Zilcha-Mano S. (2021). Toward personalized psychotherapy: the importance of the trait-like/state-like distinction for understanding therapeutic change. Am. Psychol. 76, 516–528. doi: 10.1037/amp0000629, PMID: 32658495

