# Supplementary material for: Enhancing mentalization by specific interventions within mentalization-based treatment of adolescents with conduct disorder
Source: Front Psychol. 2024 Jan 8;14:1223040. doi: 10.3389/fpsyg.2023.1223040 (PMC10800920; doi:10.3389/fpsyg.2023.1223040)
Supplement: Supplementary file 1 [file Table_1.pdf]

MBT Interventions Coding Manual

Kasper, Hauschild, Schrauf & Taubner (2023)

Intervention Level	Intervention	Characterization	Examples<br>Patient (P), Therapist (T)
Supportive & Empathic	Empathic Validation	- The therapist identifies and reflects the patient's mental state.<br>- The therapist empathises with the patient's experience.<br>- The patient's perspective is recognised as true in essence.<br>- The patient's perspective is normalised .	*"I understand, but so okay. I really understand the despair. I also understand the anger and I understand your effort not to freak out - to regulate yourself, to control yourself."*<br>*"I can understand that very well. I also found it exhausting, it was very exhausting."*
	Self-Revelation	- The therapist reveals something about herself by sharing her mental state, her affect, her thoughts regarding the patient's narrative.<br>- The therapist shows herself as a mentalizing subject.	*"So I just had the need, I have the urge to protect your mother, but that's why I wanted to know more about it, because I don't know your mother."*
	Affirmation – *in general*	- The therapist praises the patient regarding a special behavior/ attitude etc.	*"You helped your classmates – that is very friendly of you. Well done."*
	Affirmation - *Mentalization*	- The therapist identifies successful mentalizing and elaborates the positive effect on the patient.	*"You need to reach more self-awareness and you have started very well with recognizing: "I am so angry that I want to punch him in the face. I should better go somewhere else now." – that's really good!"*
	Psychoeducation / Advice	- The therapist imparts knowledge / experience.<br>- Practical advice is given.	*"Well, I think regular exercise would be very important, because that helps against depression."*
	Consent / Note	- The therapist acknowledges or agrees with what has been said.	*„Oh, okay"*
Clarification, Exploration & Challenge	Clarification	The therapist clarifies facts and events by reconstructing and contextualizing them from the patient's perspective (more on the factual level).	*"And then you got what feedback?"*
	Demand Questions	- Inquiries are made regarding the underlying cognitive mental states, both of the patient and of others.	*"Ok, do you have any idea why you pushed him, although you knew that*

			you couldn't allow yourself to do anything- regarding the risk of being expelled from school?"<br>*"And you think that's why you're miserable? How is that related?"*<br>*"Do you think that he thinks you lost it on purpose then?"*
	Paraphrase / Interpretation	- The therapist reproduces what has been said with an interpretative part.	*P: "Then mum didn't really say much about it."*<br>*T: "She did leave you alone."*
	Stop & Stand / Stop & Rewind	- The therapist identifies a situation in which the patient reacts inappropriately to interpersonal events, she slows down the pace and tries to learn more about the event step by step.	*"Okay, and I'm interested in this part. What happens there before you snap?"*
	Change of Subject	- The therapist changes the topic to a topic of higher/low involvement.	*"So Christmas was boring, okay. How was it in the family? How is it in the family at all?"*
	Challenge	- The therapist leaves the usual therapeutic dialogue with a surprising statement.<br>- The therapist uses statements that, among other things are<br>o unexpected<br>o humorous and/or teasing<br>o pointed and/or provocative<br>o strange and/or ambiguous<br>o confrontational but justified	*P: "It seems that way to me. I suppress it immediately. So when I have pain, I just make it go away inside me. They just go away like that."*<br>*T: "How do you do that, please? Can you teach me to do that?" (laughs)*
Basic-Mentalizing & Affect Mode	Offering a new perspective	- The therapist opens a new angle, a different perspective.	*"But it depends on the choice of means: you can sort of "beat them up", but without violence. I really think people take you more seriously then."*
	Mentalizing for the patient	- Underlying mental states, both of the patient and of others, are reflected and offered to the patient.	*"So they invest in you and then you also feel somehow valuable and think to yourself, yes, and now I'll really join in. Maybe that was the idea?"*
	Affect-Elaboration	- Exploration of the situation with specific focus on the patient's affect.<br>- Feelings are contextualised and related to interpersonal interactions	

		and/or significant events.<br>- Descriptions of bodily experience are stimulated to establish mental representations of affective states.<br>- mixed feelings are differentiated.	
	Demand Phrasing	- Exploration of the patient's affect, in the form of a question.	*"And then so you have a feeling about it? Can you describe that?"*
	Offering Phrasing	- Feelings are offered to help the patient identify their own feelings.	*"But I think between this "I am insecure with you" and "I need security very urgently" - in between there is somehow anger or resentment or something?"*
Relational Mentalizing	Affect focus	- The therapist names and explains the prevailing affective state at the moment, which is mutually shared by the therapist and the patient and as a factor of mutual relationship hinders the progress of the session.	*T: "But that doesn't sound bad at all, does it?"*<br>*P: "Yes it does."*<br>*T: "What is it?"*<br>*P: "You... nobody can put themselves in my shoes."*<br>*T: "No, you're right, it's actually hard for me right now."*
	Therapist-Patient-Relation	- Known interaction patterns of significant relationships are used and related to the interaction between therapist and patient<br>- The patient's behavior towards the therapist is addressed and related to patterns of relationships in everyday life.<br>- The way the patient experiences the therapist is explored and validated by naming the therapist's contribution to this experience.<br>- The therapist expresses her own feelings in order to elaborate the complexity of the patient's experience of the therapist, the understanding of the relationship as well as factors threatening the relationship.	*"But then - what just happened here between us – this pattern repeats itself inside you?"*
Additional Category			
Basic Communication	Small-Talk	- The therapist addresses issues that are not directly related to the therapeutic process.	
	Organizational	- Statements that relate to the structure of the session, such as the	

		beginning and end of the session, therapy goals, appointments.
Nonverbal	-	Non-verbal reactions of the therapist: empathic to challenging
Not Classified	-	Statements that cannot be attributed to any of the described interventions.
	-	Aborted/incomprehensible statements
